# Musical training, neuroplasticity and cognition

**DOI:** 10.1590/S1980-57642010DN40400005

**Published:** 2010

**Authors:** Ana Carolina Rodrigues, Maurício Alves Loureiro, Paulo Caramelli

**Affiliations:** 1 MSc, Neurosciences Graduation Program, Institute of Biological Sciences, Federal University of Minas Gerais, Belo Horizonte MG, Brazil.; 2 PhD, Department of Instruments and Singing, School of Music, Federal University of Minas Gerais, Belo Horizonte MG, Brazil.; 3 MD, PhD. Behavioral and Cognitive Neurology Research Group, Department of Internal Medicine, Faculty of Medicine, Federal University of Minas Gerais, Belo Horizonte MG, Brazil.

**Keywords:** musical training, neuroplasticity, cognitive abilities

## Abstract

The influence of music on the human brain has been recently investigated in numerous studies. Several investigations have shown that structural and functional cerebral neuroplastic processes emerge as a result of long-term musical training, which in turn may produce cognitive differences between musicians and non-musicians. Musicians can be considered ideal cases for studies on brain adaptation, due to their unique and intensive training experiences. This article presents a review of recent findings showing positive effects of musical training on non-musical cognitive abilities, which probably reflect plastic changes in brains of musicians.

## Abstract (pt)

A influência da música no cérebro humano tem sido alvo de vários estudos nos últimos anos. Muitos trabalhos têm demonstrado que processos neuroplásticos estruturais e funcionais surgem no cérebro como resultado de treinamento musical prolongado, os quais podem produzir diferenças cognitivas entre músicos e não-músicos. Os músicos podem ser considerados como casos ideais para investigações relacionadas à adaptação cerebral, devido às suas experiências únicas e intensivas de treinamento. Este artigo apresenta uma revisão das recentes descobertas que mostram efeitos positivos do treinamento musical em capacidades cognitivas não-musicais, o que poderia refletir a ocorrência de alterações plásticas nos cérebros de músicos.

Music seems to transcend time, space and culture and several studies are currently underway investigating the biological foundations of musical capacity, drawing on combined fields of genetics, developmental and comparative research, neurosciences and musicology.^1^ A better comprehension of the biological processes involved in musical activities may have implications not only to music making, but also to education, health care and general cultural evolution of societies.

The interest in studying the effects of musical training on cerebral function has grown considerably in the last decade. A body of evidence indicates that musicians possess structural and functional cerebral characteristics absent in non-musicians, which generally correlate to the age of commencement of musical training.^2^ Many studies^3-14^ have demonstrated changes in brains of musicians as a result of many years of musical practice. Musicians may serve as a unique model for studying plastic changes in the human brain, due to the complexity of this singular stimulus, normally related to very high levels of exposure during musical practice.^15^

The structural and functional reorganization observed in musicians’ brains may lead to cognitive differences in comparison to non-musicians. Some studies^16-28^ have shown positive associations between formal musical training in children and non-musical cognitive abilities. Moreover, other studies^29-41^ have demonstrated, directly or indirectly, enhanced cognitive abilities in adult musicians compared to non-musicians.

Our review initially presents evidence of neurobiological changes produced by long-term musical training and then focuses on the cognitive differences between musicians and non-musicians, considering visual and verbal abilities in more detail. The aim of this study was to review some interesting and relevant studies which have provided a growing body of evidence that intensive musical training may trigger neuroplastic processes, and consequently, modifications in cognitive processing. The influence of musical training on the human brain, specifically on cognition, is a recent and intriguing field of research.

## Musical training and neuroplasticity

Several investigations have demonstrated structural and functional changes, by using magnetic resonance imaging, eletroencephalography or magnetoencephalography, in brains of musicians as a result of many years of musical practice. In examining the morphometry of the corpus callosum, Schlaug et al.^4^ found that its anterior portion was significantly larger in musicians compared to non-musicians. Moreover, a comparison between subgroups revealed that this region was larger in those who began musical training earlier. The authors pointed out that the observed anatomical difference has to be taken in the context of a need for increased interhemispheric communication underlying complex bimanual motor sequences in musicians.

Amunts et al.^5^ evaluated the size of right and left motor cortices in right-handed musicians and non-musicians. As expected, a leftward asymmetry was observed in both groups, but musicians showed a smaller degree of asymmetry, since they presented a larger right motor cortex. The investigators also found a negative correlation between the size of motor cortex of both hemispheres and the age of commencement of musical practice. These authors interpreted the structural differences in motor cortex as structural compliance in response to intense and early hand skill training.

In a retrospective study, Schlaug et al.^7^ measured the volume of cerebellum in musicians and non-musicians, showing higher average relative cerebellar volume in male musicians when compared to male non-musicians, a finding interpreted as evidence for microstructural adaptations in the cerebellum in response to early commencement and continual practice of complicated bimanual finger sequences.

Gaser & Schlaug^10^ found increased gray matter volume in motor, auditory and visuospatial cerebral areas in musicians, using voxel-based morphometry analysis, a method enabling structural differences to be identified across the whole brain space. According to the authors, the results can be viewed as evidence of structural adaptations in response to long-term skill acquisition and the repetitive rehearsal of these skills, a notion supported by the strong association found by the authors between structural differences and practice intensity.

Bengtsson et al.,^11^ using diffusion tensor imaging, investigated effects of extensive piano practice on white matter in childhood, adolescence and adulthood. They found more structured right posterior internal capsule, which carries corticospinal tracts, in pianists compared to non-musicians, and also found positive correlations between practicing and fiber tract organization in different regions for each age period. The authors noted that training can induce white matter plasticity if this occurs during periods when fiber tracts involved are still undergoing maturation. Han et al.^13^ examined both gray matter and white matter in pianists and non-musicians and demonstrated, in the first group, higher gray matter density in the left primary sensory-motor cortex and right cerebellum as well as higher white matter integrity in the right posterior internal capsule. According to the researchers, these results indicate that long-term piano practicing may lead to gray matter and white matter adaptation in movement-related regions, which may impact number of synapses, volume of glia or increased myelination and diameter of axons.

Other studies have shown differences in functional cerebral characteristics between musicians and non-musicians. Elbert et al.^3^ investigated the somatosensory cortical representation of the fingers D1 (thumb) and D5 (little finger) of string players – violinists, cellists, guitarists – and non-musicians. After excitation of left-hand fingers D1 and D5, the strength of cortical activation was higher in musicians than in control subjects. This effect was particularly pronounced for finger D5, while there was no difference in cortical representations following right-hand stimulation. The authors stated that cerebral representation is enhanced to the fingers of the hand that is most intensively used in string players, and that the more a given finger is stimulated, the larger the increase in cortical response.

Pantev et al.^6^ investigated auditory cortical representation in musicians and non-musicians. After acoustic stimulation, consisting of a pseudorandom sequence of four piano tones and four pure tones matched in frequency and loudness, the strength of cortical activation for piano tones was 25% greater in musicians. There was no difference between the groups in relation to cortical representation of pure tones. The authors stressed that pure tones, unlike piano tones, are not part of our natural acoustic environment and also are not commonly encountered in musical training and practice, which may account for the observed results. Both Elbert et al.^3^ and Pantev et al.^6^ revealed a greater degree of cortical representation in musicians who began musical training early.

Koelsch et al.^8^ and Rüsseler et al.^9^ compared musicians and non-musicians by using mismatch negativity (MMN), a component of the evoked potentials in auditory cortex, obtained by submitting subjects to hundreds of identical stimuli that are randomly replaced by distinct ones, in the absence of attention to stimulus. Koelsch et al.^8^ observed MMN for slightly impure chords presented among perfect major chords, only in musicians, suggesting that they are superior in pre-attentively extracting more information out of musically relevant stimuli. Rüsseler et al.^9^ demonstrated that while musicians showed MMN for regularly spaced tones mistimed by 20 milliseconds, presence of MMN in non-musicians needed mistiming of longer than 50 milliseconds, indicating that temporal integration may be more precise in musicians.

Herdener et al.^14^ investigated plastic capabilities of the hippocampus by evaluating brain responses induced by temporal novelty in musicians and non-musicians, since the hippocampus structure, in addition to its key role for memory and spatial navigation, has been suggested to be crucially involved in various forms of novelty detection. They observed enhanced neural responses to temporal novelty in the anterior left hippocampus in professional musicians (cross-sectional study) and in music students after one year of intensive aural skills training (longitudinal study). The authors also found a correlation between hippocampal sensitivity to temporal novelty and musical abilities. They assumed that the observed changes in hippocampal activity in musicians represented a functional correlate of a tuning of aural skills, related to time interval perception, during the course of their studies.

Musacchia et al.^12^ showed that changes in functional organization also extend to subcortical sensory structures, demonstrating that musicians, compared to controls, had earlier and larger auditory and audiovisual brainstem responses to speech and music stimuli. The data also showed a positive correlation between years of musical practice and strength of brainstem response to speech stimulus, suggesting that musicians acquire an enhanced representation of pitch through musical training. Table 1 summarizes the studies cited above, which exemplify structural and functional changes in brains of musicians.

**Table 1:** Some structural and functional changes identified in musicians' brains

Brain areas	Changes	References
**Structural changes**		
Corpus callosum	Larger anterior portion	Schlaug et al.^4^
Motor cortex	Smaller degree of asymmetry between hemispheres / Increased gray matter volume	Amunts et al.;^5^ Gaser & Schlaug;^10^ Han et al.^13^
Cerebellum	Higher average relative cerebellar volume / Higher gray matter density in right cerebellum	Schlaug et al.;^7^ Han et al.^13^
Auditory cortex	Increased gray matter volume	Gaser & Schlaug^10^
Visuospatial cortex	Increased gray matter volume	Gaser & Schlaug^10^
Internal capsule	More structured right posterior internal capsule	Bengtsson et al.;^11^ Han et al.^13^
**Functional changes**		
Somatosensory cortex	Increased cortical representation of left hand fingers D1 and D5	Elbert et al.^3^
Auditory cortex	Increased cortical representation of piano tones / Mismatch negativity for subtle changes of pitch and temporal pattern	Pantev et al.;^6^ Koelsch et al.;^8^Rüsseler et al.^9^
Hippocampus	Enhanced responses to temporal novelty in the anterior left hippocampus	Herdener et al.^14^
Brainstem	Earlier and larger auditory and audiovisual responses to speech and music stimuli	Musacchia et al.^12^

All of the previously addressed studies suggested the existence of neuroplastic processes as an effect of musical training. In a longitudinal study, Altenmüller^42^ demonstrated that cortical activation during music processing reflects the auditory “learning biography”, the personal experiences accumulated over time. The author proposed a model to represent the relationship between auditory information and neural networks involved in music processing. According to his model, complexity of neural networks enhances with the complexity of auditory information. More interestingly, musical training can add mental representations of music, which may involve different cerebral substrates. These representations can be auditory, sensory-motor, symbolic, visual, among others. Therefore, according to the author, for the same level of auditory information complexity, professional musicians presumably use larger and more complex neural networks compared to non-musicians.

## Musical training and cognition

The structural and functional neuroplastic processes verified in brains of musicians may influence their cognitive functioning, and yield differences between musicians and non-musicians. Many investigators have studied the effects of extensive musical training on non-musical cognitive abilities in children^16-28^ and adults.^29-41^ The present study focuses on two cognitive domains – visual and verbal abilities – since studies comparing cognition in musicians and non-musicians have demonstrated benefits of musical training mainly in these domains. These previous studies are summarized in Table 2.

**Table 2:** Some visual and verbal cognitive changes identified in musicians and adults with music training.

Cognitive abilities	Changes	References
**Visual and verbal cognitive changes**		
Visuospatial processing	Enhanced visuospatial abilities / More balanced visuospatial attentional capacity	Brochard et al.;^31^ Patston et al.;^34^ Patston et al.;^35^ Patston et al.;^36^ Sluming et al.^62^
Oculo-motor strategies	More efficient oculo-motor strategies	Kopiez & Galley;^29^ Gruhn et al.^32^
Visual attention	Increased divided visual attention ability	Rodrigues et al.^33^
Visual processing	Enhanced visual processing of local details	Stoesz et al.^37^
Visual memory	Superior visual memory	Jakobson et al.^39^
Verbal memory	Superior verbal memory	Brandler & Rammsayer;^30^ Franklin et al.;^38^Jakobson et al.;^39^ Chan et al.;^40^ Kilgour et al.^41^

### Visual abilities

Several studies have described positive effects of musical training on visual cognition. Brochard et al.^31^ investigated visuospatial abilities in musicians and non-musicians by using a neuropsychological task in which subjects had to detect the position of a target dot relative to vertical or horizontal reference lines flashed on a screen. In one condition (perception condition), the reference line remained on the screen until the dot was displayed, while in a second condition (imagery condition), the line disappeared before the target dot was presented, requiring subjects to keep a mental image of the reference line. In both conditions musicians presented shorter reaction times compared to controls, suggesting enhanced visuospatial abilities in the former group. Another experiment in the same study compared sensory-motor abilities between the two groups, showing that visual abilities of musicians could only be partially explained by better sensory-motor integration. The investigators attributed their results to long-term practice of musical reading, which involves fine recognition of relative vertical and horizontal positions of musical notes on the score. They also ascribed the observed differences between the two groups to more efficient attentional processes in musicians.

Important aspects of music reading practice are addressed by comparing saccadic eye movements in musicians and non-musicians. According to Kopiez & Galley,^29^ the pattern of saccadic eye movements can be used as an indicator of mental disabilities, as well as a measure of mental processing speed. The same authors stated that due to specific demands of music reading, it is reasonable to presume that an early start of musical practice may modify the way visual information is processed by the nervous system of adult musicians. Kopiez & Galley^29^ and Gruhn et al.^32^ compared saccadic eye movements during oculo-motor tasks in adult musicians and non-musicians and reported more efficient oculo-motor strategies in the former, which, according to the authors, could be associated with complex visual processes involved in long-term practice of music reading. They highlighted that the difference observed between the two groups could also be related to more efficient attentional processes in musicians, although this aspect was not directly investigated by the researchers. According to Gruhn et al.,^32^ some studies^43-46^ indicate the existence of a strong association between attention and saccadic eye movements. The voluntary control of eye movements requires highly complex mental processes, involving many cerebral areas.^47^ According to Kimmig,^48^ all modalities of attention have an impact on the oculo-motor system. Fixation, characterized by a voluntary suppression of saccadic movements, as well as the rate of express saccadic movements, involve dorsolateral frontal lobe processes, which also participate in attentional mechanisms.^32^

Rodrigues et al.^33^ compared the performance of musicians, members of a symphony orchestra and a symphony band, and non-musicians, in tasks involving visual attention ability. The main neuropsychological test used in the study was the Multiple Choice Reaction Time (MCRT) test,^49^ which consisted of specific motor responses to various luminous stimuli. In order to evaluate divided visual attention ability, the MCRT test was applied twice, the second time concomitantly with other continuously and randomly changing visual stimuli presented in video form. Subjects were asked to respond verbally to each change. Musicians showed a higher percentage of correct responses to MCRT when the test was applied alone. Although no significant difference of accuracy was observed between the groups when MCRT was applied together with changing visual stimuli, musicians showed shorter reaction times for verbal responses to stimuli changes. The authors pointed out that this result may suggest augmented divided visual attention ability in musicians compared to non-musicians, which they ascribed to ensemble musical practice. The professional routine of musicians is characterized by constant demands of divided visual attention, since it requires dealing with several kinds of visual stimuli simultaneously such as the music score, conductor’s gestural instructions and body movements of other musicians, while playing the instrument.

Patston et al.^34^ compared right-handed musicians and non-musicians in a line-bisection task,^50^ which entailed marking the center of 17 horizontal lines, varying in length from 10 to 26 cm, displayed randomly on a page. As pointed by Hausmann et al.,^50^ on this task, neurologically intact right-handers show a slight yet reliable tendency to bisect about 2% to the left of the true center, a phenomenon attributed to dominance of the right hemisphere for visuospatial attention^51^. Patston et al.^34^ demonstrated that musicians showed a slight rightward bias, while non-musicians showed greater deviation to the left, and that musicians bisected the lines more accurately and with smaller intermanual difference than the control group. The researchers suggested that musicians may develop an increased ability for the left hemisphere to perform cognitive functions that are usually right-hemisphere dominant, resulting in a more balanced spatial attention.

In another study, Patston et al.^35^ investigated lateralization of visuospatial attention in musicians and non-musicians by comparing reaction times and accuracy to stimuli presented to the left and right of a vertical line, a similar task to that used by Brochard et al.^31^ in the “imagery condition”. While both groups performed more accurately with the left sided-stimuli, musicians were significantly more accurate than controls for the right-sided stimuli and also had faster reaction times overall. According to the authors, consistent with previous research,^31,34^ the results indicated a more balanced attentional capacity in musicians, as well as enhanced visuomotor ability. It is possible that musicians have an advantage on line-and-dot tasks, of the type used both in this study and that by Brochard et al.,^31^ due to their familiarity with these components from music reading. In the task used by Patston et al.,^35^ however, the line was vertical, with dots occurring to either side, removing the direct comparability of the stimuli to musical notation.

Patston et al.^36^ also studied the lateralization of visuospatial attention electrophysiologically using a measure of callosal function – interhemispheric transfer time (IHTT) – calculated by comparing the latencies of occipital N1 components of evoked potentials between hemispheres. Musicians and non-musicians responded to stimuli presented to the left and right visual fields while submitted to electroencephalography. Non-musicians showed significantly faster IHTT in the right-to-left direction than in the opposite direction and a shorter N1 latency in the left than in the right hemisphere. In contrast, musicians exhibited no directional difference between hemispheres in IHTT and no hemispheric difference in latency, indicating a more bilateral neural connectivity. The researchers proposed that bimanual training facilitates an unusual process of extra myelination that results in more balanced connections between hemispheres than normally found in those without musical training.

Stoesz et al.^37^ investigated visual processing of local details in musicians and non-musicians, by utilizing disembedding and constructional tasks. In experiment 1, they used the Group Embedded Figures Test (GEFT),^52^ which consists of presenting a series of 25 complex figures, each containing one of nine targets hidden in the design. The subject is asked to examine each test figure and to outline the hidden target as soon as they identify it. In experiment 2, the authors used two tests: the Block Design subtest from the Wechsler Adult Intelligence Scale-Third Edition (WAIS-III),^53^ that requires the subject to replicate a geometric pattern presented on a card using the top surfaces of several colored blocks, and a task involving copying possible and impossible drawings of objects. Musicians outperformed non-musicians on the GEFT, on the Block Design subtest and also on the task of copying drawings of physically impossible objects, suggesting enhanced visual processing of local details. A correlation was found between the Block Design scores and the accuracy scores for the impossible figures, but not for the possible figures, indicating that local processing ability did not correlate with drawing ability *per se*. The authors concluded that a relative strength in local processing contributed to superior performance of musicians on the drawing task. They hypothesized that the increased visual processing of local details seen in musicians may reflect training-induced changes in a fronto-parietal system involved in controlling exploratory eye movements and shifts in visual attention, skills that are important for music score reading, which also requires analysis of visual details.

Jakobson et al.,^39^ in a part of their work, studied visual memory in pianists and non-musicians by using the Rey Visual Design Learning Test (RVDLT).^54^ In this test, participants try to learn and remember a sequentially presented set of 15 line drawings of simple geometric figures, each containing two elements (e.g., a circle and a line). Recall, registered by asking subjects to draw all figures they can remember, is tested after each of five learning trials and following a delay period. A test of delayed recognition is also administered. The results suggested superior visual memory in musicians, since they outperformed non-musicians on the fourth and fifth learning trials and on the delayed recall and delayed recognition tasks. After controlling statistically for general intelligence, the group difference on the delayed recall tasks persisted. The authors emphasized that drawing ability was not a confounder in this investigation, given that all figures in the RVDLT are extremely simple. Stoesz et al.,^37^ cited above, showed that musicians and non-musicians were equally accurate at drawing possible objects. According to the researchers, the observed relationship between visual memory and musical training may be related to improvement in processes supporting attention to visual details, to increased skill of musicians at holding and manipulating visual images in working memory, which confers an advantage during the encoding proces, or may reflect superior use of high level, strategic memory processes.

Curiously, other studies examining visual memory in musicians and non-musicians^24,30,40^ have found no group differences. Brandler & Rammsayer^30^ used a task in which participants were asked to indicate, from memory, the locations of buildings on a city map they had previously studied. Chan et al.^40^ and Ho et al.^24^ used tests to assess memory for visual designs. Jakobson et al.^39^ identified some aspects in these studies that may have contributed to an apparent discrepancy. Chan et al.^40^ and Ho et al.^24^ worked with Asian samples, that would have been trained in the use of an ideographic writing system. Some previous studies^55,56^ showed that such training is associated with better memory for abstracts designs. Brandler & Rammsayer,^30^ on the other hand, did not investigate an Asian sample, but the nature of the visual memory task used by these researchers, requiring topographical skills, differed from that of Jakobson et al.^39^ Since some studies^57,58^ have suggested that neural substrates of topographical memory are separate from those supporting visual memory for faces and designs, the authors hypothesized that piano training may have a greater effect on systems involved in storing simple design information, than on those involved in storing topographical information, since music notation shares many common features with simple designs.

Some brain imaging studies have also suggested more efficient visual processes in musicians. Platel et al.^59^ showed activation of an associative visual area (Brodmann area 19) in musicians during a pitch discrimination task. The authors suggested that musicians would be able to imagine melodic lines in some kind of visual axis in order to detect pitch changes. Schmithorst & Holland^60^ investigated the relationship between musical practice and specific cerebral processing of two musical elements: melody and harmony. The results demonstrated that musicians and non-musicians recruit different neural networks to process these elements. Inferior parietal areas were activated only in musicians during melody perception (Brodmann area 40, bilaterally) and harmony perception (Brodmann area 39, left), which have been identified as involved in general visuospatial processing. Sluming et al.^61^ compared male musicians, members of a symphony orchestra, with non-musicians and revealed an increased density of gray matter in Broca’s area in the brains of the musicians. This area is an important neuroanatomical substrate for spoken language and, according to the authors, for several musically relevant abilities, including visuospatial localization. More recently, Sluming et al.^62^ showed enhanced performance on a visuospatial task by orchestral musicians, compared to non-musicians, associated with increased activation in Broca’s area.

### Verbal abilities

The skills underlying the ability to learn a piece of music resemble those involved in memorizing a poem or a piece of prose^39^. In fact, there is much evidence pointing to a link between musical practice and verbal memory ability. Most of the groups of musicians involved in such studies consisted of instrument players. Chan et al.^40^, by using a list-learning task, showed that adults who received musical training before the age of 12 for at least six years had a better memory for spoken words than those who did not. Kilgour et al.^41^ showed that young adults with musical training, when compared to controls, performed better in recalling verbal materials under all conditions, including immediate and delayed recall trials. Brandler & Rammsayer^30^ compared some mental abilities in musicians and non-musicians by utilizing a comprehensive intelligence test battery, and verified superior performance of musicians only on the verbal memory test.

Ho et al.^24^ used a hybrid cross-sectional and longitudinal design and assessed verbal memory ability in children with and without musical training using the Hong Kong List Learning Test (HKLLT).^63^ In the cross-sectional part of the study, the results showed that children with musical training demonstrated better verbal memory than those without such training. The longitudinal part of the study consisted of a one-year follow-up of the children, forming three groups: those with experience who continued lessons, those who discontinued their lessons, and those who were new to musical training. The researchers verified that children who had begun or continued musical training demonstrated significant verbal memory improvement and those who had discontinued the training showed no improvement at all. They associated their results with the neuroimaging findings of Schlaug et al.^64^ that revealed an enlargement in the left planum temporale in musicians. Ho et al.^24^ suggested that musical training during childhood may serve as a kind of sensory stimulation that contributes to the reorganization of the left temporal lobe in musicians, which in turn facilitates cognitive processing mediated by this specific brain area, as is the case of verbal memory.

Jakobson et al.,^39^ in another part of their study mentioned earlier, investigated verbal memory in pianists and non-musicians using the California Verbal Learning Test-Second Edition (CVLT-II).^65^ This test includes two aurally presented word lists, each consisting of 16 words from four different semantic categories. Recall is tested after each of five learning trials, and free and cued recalls are assessed following both short and long delay periods. The results suggested superior verbal memory in musicians, since they outperformed non-musicians on the short delay cued recall and on the long delay with both free and cued recalls. After controlling statistically for general intelligence, group difference persisted in long delay free recall trials. In line with the part of this study investigating visual memory, these results were consistent with the idea that formal music training is specifically associated with superior memory functioning, beyond any effects it may have on general intelligence. Moreover, musicians demonstrated greater use of a semantic clustering strategy during the learning phase, suggesting, according to the authors, that the verbal memory advantage does not simply reflect better memorization skills. Rather, it is associated with a relative strength in the extraction of higher-order, semantic information during encoding. The researchers highlighted that the encoding and storage of information about hierarchical relationships between words, as in the case of structural representations of music, reflects the use of strategic memory processes. As argued by Patel,^66^ even if these two types of representations are stored separately, the processes involved in activating them may depend on a common neural substrate. According to Jakobson et al.,^39^ it seems likely that changes in this neural circuit, induced by training, underlie the verbal memory advantage displayed by musicians in their study.

Franklin et al.^38^ also investigated verbal memory in musicians and non-musicians. The study was conducted in two phases: in phase 1, they tested long-term verbal memory using the Rey Auditory Verbal Learning Test (RAVLT),^67^ which uses a list-learning paradigm, and in phase 2, they introduced articulatory suppression within the same test in order to interfere with verbal rehearsal processes. As described by the researchers, articulatory suppression, achieved by giving subjects the additional instruction of saying the word “the” between each word read from the list, is a technique that prevents participants from rehearsing verbal material to aid its storage in working memory and after in long-term memory. They also tested verbal working memory using reading-span and operation-span tasks. The results showed that musicians outperformed non-musicians in phase 1, but not in phase 2. According to the authors, the fact that the superior performance of musicians is lost with the introduction of articulatory suppression may suggest that an overt rehearsal strategy is being used by musicians to boost performance. Moreover, a greater verbal working memory capacity for musicians was also found. Therefore, the researchers suggested that musical training affects verbal long-term and working memory and that enhanced rehearsal mechanisms are likely responsible for superior performance of musicians in verbal memory tasks.

Results of some brain imaging studies have been consistent with more efficient verbal processes in musicians. Schlaug et al.,^64^ cited earlier, investigated the morphometry of the planum temporale, a brain area containing auditory association cortex and previously shown to be a marker of structural and functional asymmetry, and found stronger leftward asymmetry in musicians compared to non-musicians. The researchers considered this asymmetry as potentially related to language and pitch processing skills. Ohnishi et al.^68^ assessed cortical activation during a passive listening task and found greater activation of the planum temporale and the left dorso-lateral prefrontal cortex in musicians than in controls. The authors also found a negative correlation between the degree of activation in the left planum temporale and the age at commencement of musical training. Some studies^69-71^ have shown that musicians often demonstrate greater left-hemisphere lateralization than non-musicians when presented with musical stimuli. More recently, Fujioka et al.^72^ investigated brain activation in children while listening to violin tones and also observed a greater left-hemisphere lateralization in those children who received music lessons throughout a year.

## Final comments

An important issue related to music and cognition that remains unresolved, as emphasized by Schellenberg & Peretz,^73^ is the question of causation. It is not yet known whether enhanced cognitive abilities verified in musicians are in fact a consequence of long-term training or are innate. Most of the studies suggesting benefits of musical practice on cognition are of a correlational or quasiexperimental nature, hampering the determination of a clear causal link.

However, some studies have provided evidence supporting the idea of training-related effects of music on brain and cognition. For instance, training studies,^18,20,22^ using a pre-test and post-test design, have demonstrated augmented spatial abilities in children after periods of music lessons. It has also been reported, as mentioned previously, that the degree of structural and functional enhancement in specific brain areas correlates with the age of onset of musical training,^3-6,68^ the number of years of musical practice^12,61^ and its intensity.^10,11^ Norton et al.,^74^ in a study involving children aged five to seven years, compared those who were about to begin music studies with those who were not, and found no pre-existing neural, cognitive, motor or musical differences between the groups. Part of an ongoing longitudinal investigation into the effects of musical practice on brain and cognitive development, initiated by Norton et al.,^74^ Hyde et al.^75^ demonstrated regional structural brain plasticity that occurred after only 15 months of musical instrument training in early childhood. Structural brain changes in motor and auditory areas were correlated with behavioral improvements on motor and auditory-musical tests. According to the researchers, the lack of brain and behavioral differences between music trainees and control children at baseline supported the view that differential brain development is induced by intensive musical training rather than by preexisting biological predictors of musicality.

It is also noteworthy that musical practice is just one sort of training that can lead to neural and cognitive changes on human brain. Other studies have demonstrated such changes after different forms of training. For example, Maguire et al.^76^ reported that taxi drivers have larger posterior hippocampal volume in comparison to the general healthy population, argued to be due to the intense visuospatial demands of their job. Draganski et al.^77^ observed that adults trained for three months in juggling showed an increase of gray matter in particular brain areas, followed by a decrease after a three-month cessation of juggling. Pascual-Leone et al.^78^ showed that the motor cortical output map to the reading hand in Braille readers was even modifiable after only a few hours of training. Similarly, Karni et al.^79,80^ have shown enhanced activation of the primary motor cortex following daily practice of a finger opposition task. Sims & Mayer^81^ demonstrated enhanced mental rotation skills in video game players. It has been shown that children (Carlson & Meltzoff^82^), adults (Colzato et al.^83^) and older adults (Bialystok et al.^84^) who are lifelong bilinguals and use both languages on a regular basis exhibit higher levels of executive control than comparable monolinguals.

Hence, several forms of intensive training have an impact on brain and cognition, but it is possible that musical training has specific effects that other forms of training do not have, or even produces a range of different effects, considering its multifaceted nature. According to Norton et al.,^74^ cognitive enhancement effects of musical training, the result of neuroplastic processes, might be due to a combination of skills required by music study, such as decoding visual information into motor activity, memorizing extended passages of music, learning music structures and rules, learning to make fine auditory spectral and temporal discriminations and learning to perform skilled bimanual finger movements.

As emphasized by Stoesz et al.,^37^ studies demonstrating enhanced cognitive abilities in musicians can be of interest on both theoretical and applied grounds. At a theoretical level, such studies may lend a better understanding of the effects of musical practice on the development and functioning of multiple neural systems, which support a range of cognitive abilities, whereas at an applied level, they may contribute to advances in the fields of education and cognitive rehabilitation.
